# Arctic Ocean Microbial Community Structure before and after the 2007 Record Sea Ice Minimum

**DOI:** 10.1371/journal.pone.0027492

**Published:** 2011-11-09

**Authors:** André M. Comeau, William K. W. Li, Jean-Éric Tremblay, Eddy C. Carmack, Connie Lovejoy

**Affiliations:** 1 Québec-Océan, Département de Biologie, and Institut de Biologie Intégrative et des Systèmes (IBIS), Université Laval, Québec, Québec, Canada; 2 Fisheries and Oceans Canada, Bedford Institute of Oceanography, Dartmouth, Nova Scotia, Canada; 3 Fisheries and Oceans Canada, Institute of Ocean Sciences, Sidney, British Columbia, Canada; Argonne National Laboratory, United States of America

## Abstract

Increasing global temperatures are having a profound impact in the Arctic, including the dramatic loss of multiyear sea ice in 2007 that has continued to the present. The majority of life in the Arctic is microbial and the consequences of climate-mediated changes on microbial marine food webs, which are responsible for biogeochemical cycling and support higher trophic levels, are unknown. We examined microbial communities over time by using high-throughput sequencing of microbial DNA collected between 2003 and 2010 from the subsurface chlorophyll maximum (SCM) layer of the Beaufort Sea (Canadian Arctic). We found that overall this layer has freshened and concentrations of nitrate, the limiting nutrient for photosynthetic production in Arctic seas, have decreased. We compared microbial communities from before and after the record September 2007 sea ice minimum and detected significant differences in communities from all three domains of life. In particular, there were significant changes in species composition of Eukarya, with ciliates becoming more common and heterotrophic marine stramenopiles (MASTs) accounting for a smaller proportion of sequences retrieved after 2007. Within the Archaea, Marine Group I *Thaumarchaeota*, which earlier represented up to 60% of the Archaea sequences in this layer, have declined to <10%. Bacterial communities overall were less diverse after 2007, with a significant decrease of the *Bacteroidetes*. These significant shifts suggest that the microbial food webs are sensitive to physical oceanographic changes such as those occurring in the Canadian Arctic over the past decade.

## Introduction

Atmospheric and oceanic processes are directly affected by increasing global temperatures and the Arctic has been the most severely impacted region to date [**1**]. A long-term trend of deceasing minimum extent of summer sea ice was marked by a sharp decline and record low in September 2007. Summer sea ice extent remains below the long-term pre-2007 average [**1]**, [2****]. While direct temperature effects appear to be the main driver of changes in terrestrial biomes, there is little data on whether and how physical changes impact the underlying microbial food webs that support all higher marine trophic levels [**3**]. Upper Arctic waters have warmed, but also freshened since the 1990s due to the melting of multiyear sea ice, increasing river runoff and increased transport of surface Pacific Water into the Arctic Basin [**2]**, [4****]. Increased stratification of the upper water column due to surface freshening could have a profound effect on nutrient transport into the euphotic zone and decrease the overall productivity of the Arctic Ocean. Evidence for such a change has been reported from the upper 150 m of the Canada Basin, where smaller phytoplankton size-classes are becoming more prevalent [**5**].

The Arctic is much more quiescent than any other ocean because of salinity stratification and the duration of both multiyear and seasonal ice cover [**6**]. A persistent feature of this strong stratification is a subsurface chlorophyll maxima (SCM) layer that tracks the interface of Pacific Summer Water (PSW) and the Arctic Winter Water (AWW). The SCM is estimated to account for 70% of annual photosynthetic production in the Amundsen Gulf region of the Beaufort Sea (J Martin and J-E Tremblay, unpublished modelling result) and is a persistent feature throughout the Arctic Ocean. Protist communities within this layer change little over geographical space despite changes in depth [**7**] and at least one isolate from the Arctic has a remarkable capacity to adjust to varying irradiance levels [**8**]. Similarly, bacterial and archaeal taxa change little within distinct water masses [**9]**, [10****]. However, interannual variation of communities within a given water-mass has not been systematically investigated. The principal goal of this study was to investigate whether the physical and nutrient properties of PSW had changed substantially since 2007 following the massive loss of summer sea ice and whether any changes were accompanied by changes in SCM microbial communities. To fulfill our aim, we analyzed available physical and nutrient data from multiple oceanic missions to the Amundsen Gulf region from 2002–2010, and available microbial DNA samples collected from 2003–2010 from the SCM, where photosynthetic activity occurs from June though early November [**11**]. All three domains of life were targeted using a high-throughput, tag small-subunit (SSU) rRNA gene amplicon sequencing approach [**12**]. To maximize the microbial signal we focused on the 0.2–3 µm nominal size-fraction which enriches for smaller cells [**13]**, [14****]. Since size-fractionation by filtration is inefficient, this approach provides comparative information on larger cells as well.

## Materials and Methods

### Physicochemical sample collection and processing

Water samples, conductivity, temperature and depth (CTD; Sea-Bird SBE­911) profiles were taken during missions (2002–2010) to the Amundsen Gulf, Beaufort Sea region using a rosette system aboard the *CCGS Amundsen*. Additional CTD and nutrient data were collected in 2007 aboard the *CCGS Louis St. Laurent* and 2008 from the *CCGS Sir Wilfred Laurier*. Water samples were collected for nutrient analysis every 10 m to 100 m and additional samples taken at the depth of the SCM and the nitracline. These were detected on the downward cast from the output of the fluorometer (SeaPoint) for chlorophyll (Chl) and an *in situ* ultraviolet spectrometer (ISUS) for relative nitrate concentrations (Satlantic MBARI-ISUS). Irradiance as photosynthetically-active radiation (PAR) was estimated *in situ* with a PAR sensor (Biospherical Instruments) also mounted on the rosette. Salinity values from the CTD profiles were calibrated using a salinometer (Guideline Model 8400B). Samples for nutrient determinations were collected into acid-cleaned polyethylene tubes after thorough rinsing and pre-filtration; stored at 4°C in the dark and analyzed within a few hours for NO_2_^−^, NO_3_^−^ + NO_2_^−^, PO_4_^3−^ and Si(OH)_4_ using standard colorimetric methods adapted for the AutoAnalyzer 3 (Bran+Luebbe).

To characterize the change of Pacific-origin water in central Amundsen Gulf, we extracted salinity and nutrient data collected from stations deeper than 300 m water depth in ice-free months (July, Aug., Sep., Oct.) for all years. The number of stations included in the analysis varied by year as follows: 2002 (*n* = 10), 2003 (*n* = 16), 2004 (*n* = 14), 2005 (*n* = 4), 2006 (*n* = 16), 2007 (*n* = 13), 2008 (*n* = 29), 2009 (*n* = 40), and 2010 (*n* = 16). The time-series trends were calculated by linear regression of year-based station averages of samples within the salinity range of 31–33, approximating the water mass defined by the T_max_ of the PSW, and the T_min_ of the Pacific Winter Water, which lies just below the PSW [**15**]. Chlorophyll *a* (Chl *a*) samples were collected, filtered and analyzed on board using standard techniques as previously reported [**16**].

Arctic Ocean sea ice extent data was obtained from the IARC-JAXA website (http://www.ijis.iarc.uaf.edu/en/home/seaice_extent.htm) and the 30 daily values for September (month of minimum yearly extent) were averaged as a representation for each year from 2003–2010.

### Biological sample collection and processing

**Figure 1:**
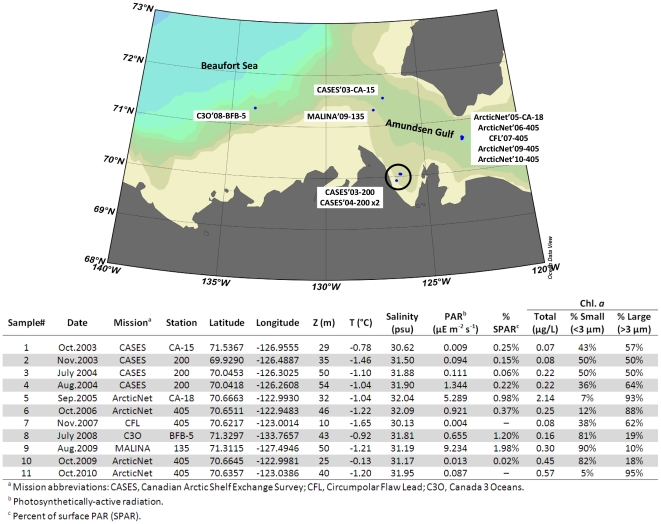
Sampling sites and dates. Locations, physicochemical parameters, and chlorophyll a concentrations of the samples used for tag pyrosequencing.

Samples were collected from the Amundsen Gulf, Beaufort Sea, between October 2003 and October 2010 (**Fig. 1**) in conjunction with 8 different research expeditions aboard the *CCGS Amundsen* (all years, except 2008) and *CCGS Sir Wilfred Laurier* (2008). The SCM forms in the Amundsen Gulf following the spring bloom and, although surface nitrate concentrations are near detection limits throughout summer, concentrations of nitrate in PSW remain relatively high. Since the aim was to compare SCM communities from different years in the Amundsen Gulf, samples were selected to represent typical post-spring-bloom, light-limited communities; in addition, sufficient biomass for amplification was needed. Two samples met this criteria for three (2003, 2004, 2009) of the eight years, but only one sample for each of the other years was available. Five to 7 liters of seawater were collected as previously described [**7**] from the SCM. Filters were preserved at −80°C until DNA was extracted using lysozyme, proteinase K, SDS and a salt-based separation as previously described [**17**].

### PCR primer design for 454 pyrosequencing

PCR primers targeting the SSU rRNA gene within the 16S V6-8 region of Bacteria and Archaea, and the 18S V4 region for Eukaryotes, were adapted from existing rRNA primers and designed *de novo* to ensure amplicon sizes appropriate for 454-Roche™ chemistry (**Table S1**). Care was taken to design 95% consensus primers, as determined using ARB (http://www.arb-home.de/) with the SILVA 100-SSU-Ref database, and their specificities were checked against the RDP database (http://rdp.cme.msu.edu/). Forward primers included Roche's A adaptor and MIDs (“multiplex identifiers”) in the form of: 5′-[A-adaptor]+[MID1 to 10]+[specific F primer]-3′; reverse primers included Roche's B adaptor in the form of: 5′-[B-adaptor]+[specific R primer]-3′. Full sequences of all 39 primers are listed in **Table S2**. Final amplicon lengths were 508 bp for the Bacteria and Eukarya, and 516 bp for the Archaea.

### SSU rDNA gene amplification and 454 pyrosequencing

Extracted DNA from the 0.2–3 µm size-fraction was amplified in three independent 50 µL aliquots per domain using full-length 454 primers. PCR reactions contained: 1X HF buffer (NEB), 200 µM of each dNTP (Feldan Bio), 0.4 mg/mL BSA (Fermentas), 0.2 µM of each 454 primer (Invitrogen), 1 U of Phusion High-Fidelity DNA polymerase (NEB), and 1–3 µL of template DNA. Three separate DNA concentrations were used for each sample: 1/0.5/0.1X for Bacteria and Eukarya; and between 3X and 0.1X for Archaea that represent the smallest proportion of microbial biomass in the Beaufort [**18**]. Cycling conditions were as follows: an initial denaturation at 98°C for 30 s, followed by 30 cycles of denaturation at 98°C for 10 s, annealing at 55°C for 30 s, extension at 72°C for 30 s, and a final extension at 72°C for 5 min. The triplicate reactions for the separate domains were pooled, purified using the QIAquick PCR purification kit (QIAGEN), and quantified spectrophotometrically (Nanodrop ND-1000). The 11 sample-coded amplicons were mixed in equal quantity and 1/8^th^ plate for each domain was sequenced on a Roche 454 GS-FLX Titanium platform at the McGill University/Génome-Québec Innovation Centre for Bacteria and at the IBIS/Université Laval Plate-forme d'Analyses Génomiques for Archaea and Eukaryotes. The raw pyrosequencing reads have been deposited in the NCBI Sequence Read Archive with accession number SRA029114 and a “Minimal Information about a MARKer gene Sequence” (MIMARKS) compliant table is included with the data and given here in **Table S3**.

### Pyrotag pre-processing and quality control

Raw 454 reads were processed to remove low-quality reads defined using the following criteria: (i) presence of uncertain bases, one or more Ns; (ii) short reads, reads <150 bp after adaptor and MID removal; (iii) unusually long reads, reads of greater than expected amplicon size; and (iv) reads with incorrect F primer sequence. Reads were also trimmed of all bases beyond the R primer. These quality-control steps have been shown to reduce 454 sequencing error rates to <0.2% [**19**] without the need for more involved “denoising” applications [**20**] that can be computationally prohibitive and whose theoretical assumptions are not universally accepted [**21**]. The above, high-quality “final reads” were then aligned by domain using Mothur [**22]**, [23****] (http://www.mothur.org/) against the provided SILVA reference alignments using the ksize = 9 parameter. The resulting alignments were manually refined by removing those reads that were misaligned, generating the high-quality “final aligned reads” used for all downstream analyses (**Table S4**). The number of input reads for each bar-code was also randomly re-sampled from the total reads available to have the same number in all bar-codes, equal to the lowest number present in any one bar-code resulting in: 2474 per sample for Bacteria, 8700 per sample for Eukarya and 1118 per sample for Archaea.

### OTU and taxonomy analyses

The final aligned reads were clustered into Operational Taxonomic Units (OTUs) at the ≥97% similarity level for Bacteria and Archaea and at the ≥98% similarity level for Eukarya using Mothur (furthest-neighbor clustering). The former approximates a common species definition for prokaryotes, while the latter is a compromise between the preferred ≥99% species level and the recent recommendation for using a maximum of 98% with 454 GS-FLX Titanium chemistry to avoid misidentifying V4 Eukarya tags [**21**]. Singletons, OTUs comprised of single sequences occurring only once in the datasets, were removed at this step.

Measures of diversity, rarefaction, and community similarity analyses (shared OTUs and Bray-Curtis trees) were carried out in Mothur. Bacterial OTUs were taxonomically identified using the Classifier tool of the RDP database [**24**] using the 50% bootstrap cut-off value that has been shown to be >95% accurate at the genus level [**25**], even for V6 amplicons that are much shorter than ours (∼80 bp). Archaea and Eukarya OTUs were taxonomically identified within Mothur (using 50% bootstrap cut-off) using user-designed reference sequence databases and taxonomy outlines (both available upon request), based upon a modification of the NCBI taxonomies and included our curated Arctic-specific sequences. Common “unclassified OTUs” generated from the above techniques were further identified using BLASTn at the NCBI. Fungi, Metazoa (except Choanoflagellates) and Streptophyta (plants) were ignored for the Eukarya, as were chloroplasts for the Bacteria.

### Statistical analyses

Because of limited ship time and logistic constraints on multidisciplinary missions, sampling was not carried out at a single site at the same time of the year for all the years – there is no routine monitoring of the Canadian Arctic at this time or previously. We therefore tested the significance of confounding factors that could potentially influence community structure. In addition to ice extent before and after 2007 and time (year), we tested for significant effects due to: 1) geography, notably Franklin Bay, which is less directly linked to dominant current systems, versus offshore Amundsen Gulf; 2) season, by comparing summer (July, August and Sept) versus autumn (Oct and Nov) samples; 3) irradiance as PAR levels binned as <0.1 µE m^−2^ s^−1^, 0.1–1.0 µE m^−2^ s^−1^ and >1.0 m^−2^ s^−1^ at the depth sampled; and 4) the community size distribution estimated from Chl *a* concentrations of small (0.7–3 µm) and large (>3 µm) fractions of filtered biomass. The percent distribution of Chl *a* in the small versus large fractions was taken as a proxy for successional stage, since larger cells are favoured when growth conditions are more favourable. Communities were binned as >60%, 40–60%, or <40% in the large fraction. Details of the distribution of all samples into the above categories are given in **Table S5**. Differences between two categories were tested with either the two-sample *t*-test (normal distributions) or Mann-Whitney test (non-normal distributions). Trends with three categories were tested using the Kruskal-Wallis test (non-parametric version of ANOVA). The temporal trends over 8 years, 2003–2010, were tested using linear regression (normal distributions) or the Spearman Rank correlation (non-normal distributions). Correspondence analysis (CA) was performed on the taxonomic distribution data at the level showing the most significance: phylum-level for Bacteria, major group-level for Eukarya, and “group” equal to genus-level for Archaea that have inherent reduced taxonomic complexity at higher levels. These ordination plots and all statistical analyses were carried out in PAST (http://folk.uio.no/ohammer/past/).

## Results

### Physical changes in the Amundsen Gulf and Beaufort Sea

**Figure 2:**
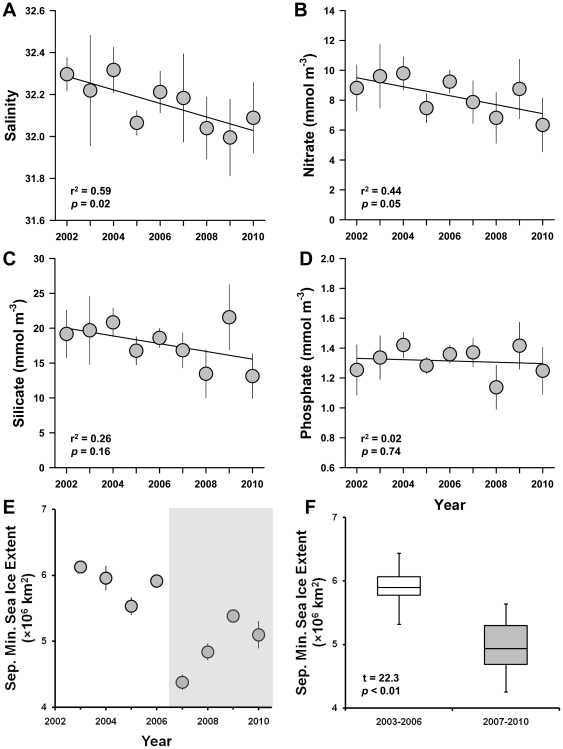
Physicochemical trends in the Arctic Ocean. (**A–D**) Salinity and nutrient concentrations in the central Amundsen Gulf. Values are from within the salinity layer bounded by 31 and 33 psu, approximating the Pacific water mass defined by the T_max_ of the summer water and the T_min_ of the winter water [**15**]. Values are averaged over all stations in waters deeper than 300 m; station variability is indicated by standard deviation. Time series trend is indicated by linear regression of year average, *n* = 9. (**E–F**) September minimum sea ice extent in the Arctic Ocean from the IARC-JAXA. Values for each year (**E**) are the averages of the 30 daily values of September and variability is indicated by standard deviation. The boxplot (**F**) indicates the medians, 1^st^ and 3^rd^ quartiles, and ranges (whiskers) of the data in (**E**) for the 4 years before the major melt of 2007 and the four subsequent years (*n* = 120 for each case). The statistical result indicated is the two-sample *t*-test (unequal variance).

The data compiled from stations greater than 300 m maximum depth over the study region indicated that Pacific waters reaching the Amundsen Gulf had freshened consistently since 2002 (**Fig. 2A**). Overall nitrate values in this layer had also significantly decreased with average values of 9.4 mmol m^−3^ from 2002–2004 to a minimum value of 6.5 mmol m^−3^ 2010 (**Fig. 2B**). Silica concentrations, while trending downward, did not change significantly (**Fig. 2C**). No change in phosphate concentrations were detected (**Fig. 2D**). Minimum annual sea ice extent occurred in 2007 and remained below the previous long-term average for the following 4 years (**Fig. 2E**), as has been extensively reported elsewhere [**26**], including over the Beaufort Sea and Amundsen Gulf region [**2**].

### Pyrosequencing and analysis overview

The numbers of final reads (and average length) following quality filters was: 44,287 (386 bp) for Bacteria, 74,775 (382 bp) for Archaea and 173,549 (402 bp) for Eukarya (**Table S4**). Once reads were re-sampled and singleton OTUs removed, all three domains showed saturation in the rarefaction analysis, with the Bacteria roughly twice as diverse as the Archaea (both at the 97% similarity level) and the Eukarya representing near 12,000 OTUs at the 98% level (**Fig. S1**). Samples were then grouped (**Table S5**) to test for the influences of geography (Franklin Bay [*n* = 3] vs. offshore Amundsen Gulf stations [*n* = 8]), season (summer [*n* = 5] vs. fall [*n* = 6]), PAR at the time of sampling (binned as high [*n* = 3], medium [*n* = 3] and low [*n* = 5]) and size-fractionated Chl *a* results (binned as >60% [*n* = 5], 40–60% [*n* = 3], and <40% [*n* = 3] of the Chl *a* in the large fraction).

We tested the likelihood that the above factors, apart from before and after the 2007 ice minimum, may have influence our assessment of community changes using two different methods. Firstly, we constructed correspondence analysis ordination plots (**Fig. S3**) based upon the proportions of the different groups in each sample at different taxonomic levels. Secondly, Bray-Curtis similarity trees were constructed based on total OTU composition in the samples at the level of individuals, independent of taxonomic assignments, where samples that group together share more OTUs than those further apart. The taxonomy ordination plots showed relatively clear patterns along the primary axes with separation for the influence of ice extent, with only one 2003 sample, not grouping with the earlier years (**Fig. S2**). The OTU Bray-Curtis topologies showed similar separation, with one sample each for Bacteria and Eukarya not consistent with the before-and-after 2007 categorization. The Archaea communities were well segregated and the before-and-after sea ice minimum samples showed no overlap. These Bray-Curtis trends can be tested statistically since the existence of a cluster in the tree implies that the numbers of shared OTUs are higher among those members of the cluster than with members outside the cluster, anywhere else in the tree. Therefore, we tested for a difference between the numbers of shared OTUs within the low ice (<2007) and high ice (>2007) clusters compared to between the two clusters. Significance was found for the Bacteria for this before-and-after 2007 grouping (*p* = 0.04), and for a linear decrease of shared OTUs over time (*p*<0.01). Similarly, trends were significant for Archaea for before-and-after 2007 (*p* = 0.03) and for the linear decrease over time (*p*<0.01). With the exception of a geographic influence on the Archaea (*p* = 0.02), no significant effects were uncovered after segregating the OTUs into the other categories tested: season, PAR, and Chl *a* size distribution. Finally, there was no statistical support for the Eukarya topologies.

### Overall community diversity

**Figure 3:**
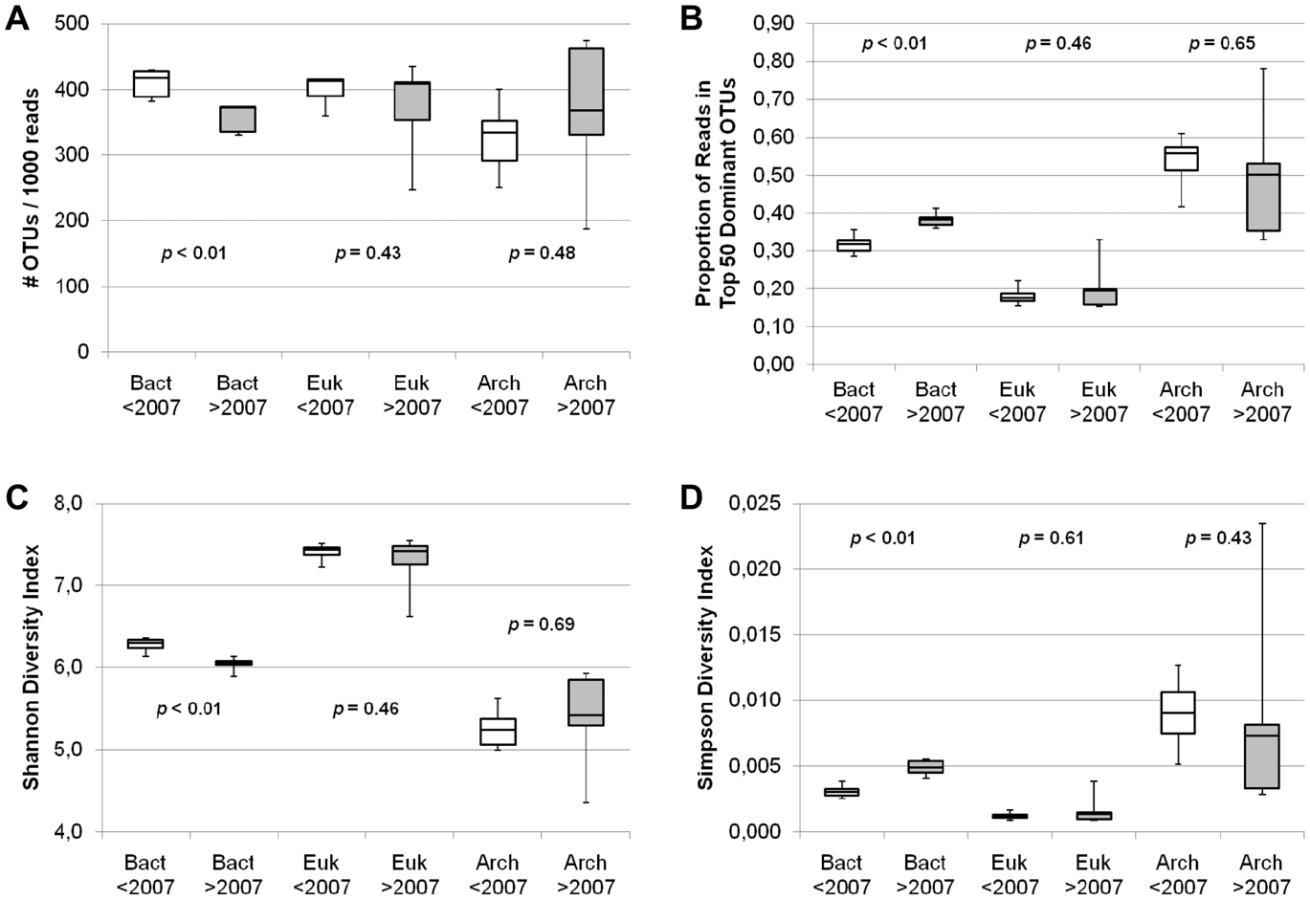
α-Diversity measures for Bacteria (97% OTU level), Eukaryotes (98% OTU level) and Archaea (97% OTU level). The boxplots indicate, as in Fig. 2, the medians, 1^st^ and 3^rd^ quartiles, and ranges (whiskers) of the data from 2003–2006 (<2007; *n* = 6) vs. 2007–2010 (>2007; *n* = 5) for Bacteria (Bact), Eukaryotes (Euk) and Archaea (Arch). The statistical results indicated are from either the two-sample *t*-tests (normal data) or Mann-Whitney tests (non-normal data). (**A**) Total richness (OTUs) per 1000 reads (to normalize for bar-code differences). The Bacteria also show a significant linear decrease (r^2^ = 0.75, *p*<0.01) over the 8 years. (**B**) Proportion of reads in top 50 dominant OTUs. The Bacteria also show a significant linear increase (r^2^ = 0.82, *p*<0.01) over the 8 years. (**C**) Shannon diversity index. The Bacteria also show a significant linear decrease (r^2^ = 0.82, *p*<0.01) over the 8 years. (**D**) Simpson diversity index. Note that the index should behave in the opposite direction of the other indices since it nears 0 as diversity increases. The Bacteria also show a significant linear increase (r^2^ = 0.86, *p*<0.01) over the 8 years.

**Figure 4:**
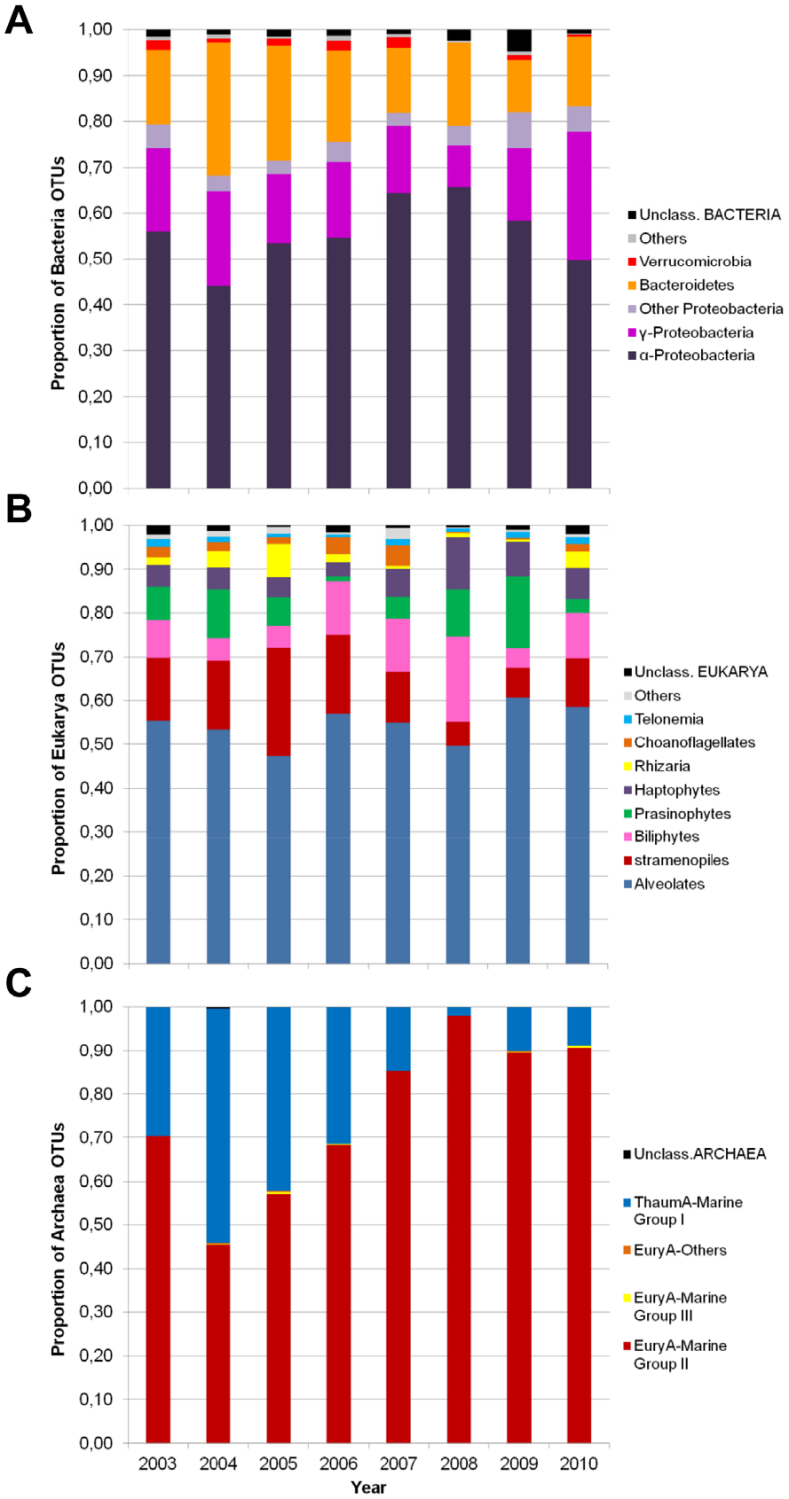
Taxonomic distribution of all OTUs. (**A**) Distribution of major Bacteria phyla and orders (97% level). (**B**) Distribution of non-Metazoan (retaining Choanoflagellates) Eukarya major groups (98% level). (**C**) Distribution of Archaea groups (97% level). ThaumA, *Thaumarchaeota*; EuryA, *Euryarchaeota*.

Bacterial richness, based on the total number of OTUs, declined significantly after 2007 with total diversity also affected (**Fig. 3**). The Shannon and Simpson indices showed significant changes in community evenness; there were fewer rare OTUs and more high frequency ones, defined as the top 50 OTUs representing the most sequences (**Fig. 4B**). Additional regression analysis indicated that the linear decrease in richness and evenness were statistically significant (**Table 1**). In contrast Archaea and Eukarya diversity indices showed no temporal trends or significant differences for before and after 2007. The potential influence of coastal samples (geography) on diversity indices was investigated by removing Franklin Bay samples. This reanalysis did not affect the significant levels for the Bacteria; Archaea and Eukarya results were also unchanged. The remaining factors (season, PAR, proportion of Chl *a*) did not significantly influence the diversity indices among the three domains (**Table 1**).

**Table 1:** Comparison of the significant trends (*p* ≤ 0.05) when analyzed for 6 different factors.

	Analysis Factor					
Trend	Ice Extent	Geography	Year	Season	PAR	Chl *a* Distrib.
***Diversity Measures***						
Shannon index – Bacteria	**−4%**	ns	**−0.91**	ns	ns	ns
Simpson index – Bacteria	**+58%**	ns	**0.92**	ns	ns	ns
***OTU Distributions***						
# OTUs – Bacteria	**−12%**	ns	**−0.81**	ns	ns	ns
# OTUs/1000 reads – Bacteria	**−15%**	ns	**−0.86**	ns	ns	ns
% reads in dominant OTUs – Bacteria	**+21%**	ns	**0.91**	ns	ns	ns
# shared OTUs (within vs. between groups) – Bacteria	≠	ns	**−0.36**	ns	ns	ns
# shared OTUs (within vs. between groups) – Archaea	≠	≠	**−0.42**	ns	ns	ns
***Taxonomic Distributions – Bacteria***						
*Bacteroidetes* (*Flavobacteria*)	**−65%**	ns	ns	ns	ns	ns
*Pelagibacter*	ns	**FB < Ocean**	ns	ns	ns	ns
***Taxonomic Distributions – Eukarya***						
Ciliates	**+53%**	ns	ns	ns	ns	ns
Haptophytes	**+71%**	ns	ns	ns	ns	ns
Rhizaria	**−62%**	ns	ns	ns	ns	ns
Stramenopiles	**−51%**	ns	ns	ns	ns	ns
***Taxonomic Distributions – Archaea***						
*Euryarchaeota*-Marine Group II	**+50%**	**FB < Ocean**	**0.80**	ns	ns	ns
*Thaumarchaeota*-Marine Group I	**−77%**	**FB > Ocean**	**−0.81**	ns	ns	ns

Trends with two categories were tested using either the *t*-test (normal data) or the Mann-Whitney test (non-normal data): Ice Extent (<2007 vs. >2007), Geography (Franklin Bay [FB] vs. open-ocean stations), Season (summer vs. fall) and searching for a difference within vs. between groups for the # shared OTUs. Trends with three categories were tested using the Kruskal-Wallis test (non-parametric version of ANOVA): Photosynthetically-Active Radiation (PAR; low vs. medium vs. high) and Chl *a* Distribution (picoplankton-dominated vs. shared vs. nanoplankton-dominated). The temporal trends (over 8 years, 2003–2010) were tested using linear regression (normal data) or the Spearman Rank correlation (non-normal data). Only those trends showing statistical significance out of the parameters tested are shown. Differences are indicated as either: overall percent increases (+X%) or decreases (−X%) for the two Ice Extent categories; FB more or less abundant than open-ocean for Geography; increasing or decreasing linear trends (*r* value) over the 8 Years; or “ns” for non-significant. For the # of shared OTUs it is a little different: using the example of Ice Extent, each sample from <2007 is compared to the other samples from <2007 (# shared OTUs within the group) and then to the samples from >2007 (between the groups). If the community structure is the same, they should share the same # of OTUs within vs. between groups. However, if there is a significant difference (**≠**), then there has been a change in structure. For the linear decreasing trends, this implies that the more years separating samples, the less OTUs they share – indicating change in community structure over time. See Supplementary Table S5 for the assignments of the 11 samples into each of the respective categories.

### Specific taxonomic changes

**Figure 5:**
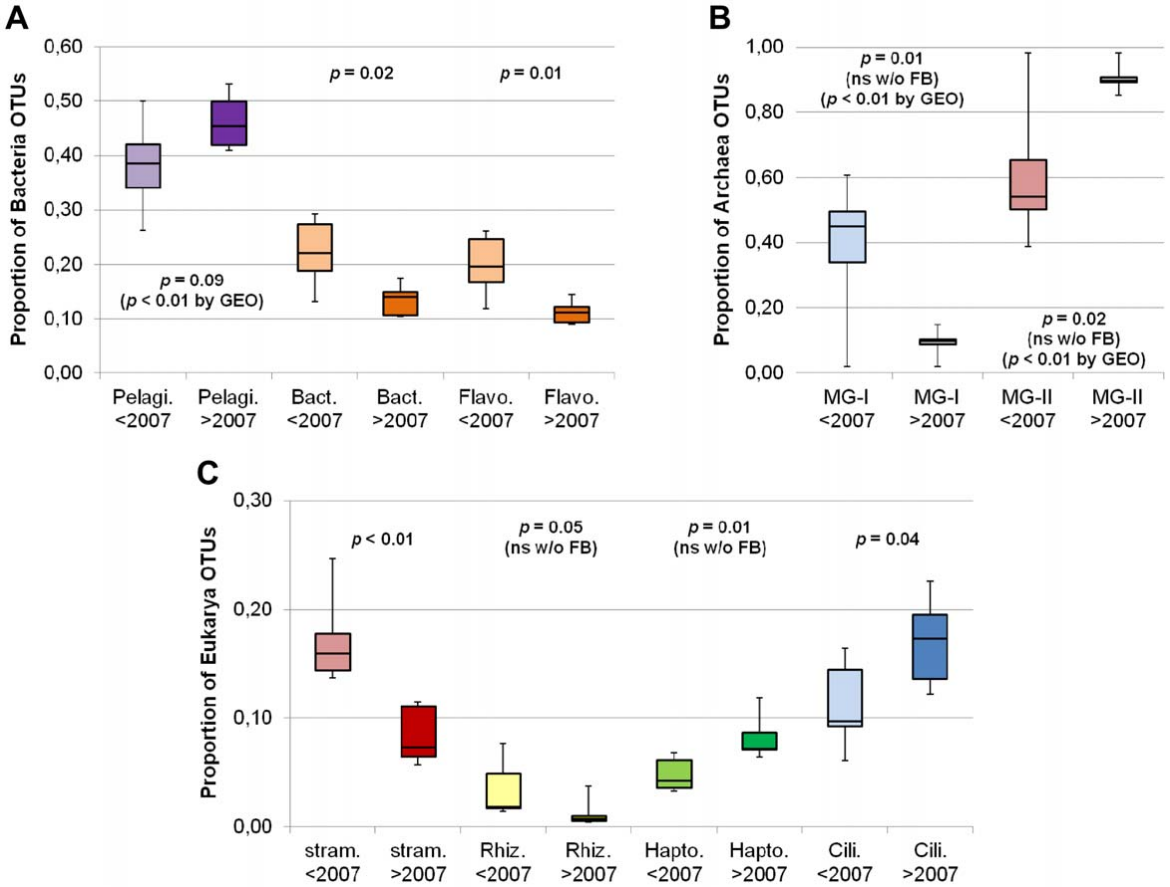
Taxonomic groups showing changes. Boxplots of select taxonomic groups, with parameters as in Fig. 3 and colors corresponding to Fig. 4. Lighter shades indicate 2003–2006 and darker shades indicate 2007–2010. Those parameters that became non-significant (ns) when the coastal Franklin Bay (FB) samples were removed are indicated. Conversely, those that became significant when analyzed geographically (GEO; coastal Franklin Bay vs. open-ocean sites) are also indicated. (**A**) Various bacterial taxa (97% level). Pelagi., *Pelagibacter* spp.; Bact., *Bacteroidetes* phylum; Flavo., *Flavobacteria* class. (**B**) Archaea groups (97% level). The MG-I and MG-II show significant linear trends (r^2^ = 0.65, *p* = 0.02 and r^2^ = 0.64, *p* = 0.02) over the 8 years. (**C**) Various Eukarya taxa (98% level). Stram., stramenopiles; Rhiz., Rhizaria; Hapto., Haptophytes; Cili., Ciliates.

*Alphaproteobacteria* consistently represented the largest proportion of OTUs (**Fig. 4A**), the majority of which (78%) were *Pelagibacter* (the SAR11 clade). Among the dominant OTUs (**Fig. S4**), *Pelagibacter* was the also the most abundant, sometimes representing over 25% of all bacterial sequences. The *Gammaproteobacteria* accounted for 10–20% of the bacterial OTUs, with little change over time. Among the other major bacterial groups, the proportion of *Bacteroidetes* sequences decreased prior to 2007 (**Fig. 4A**) and were significant fewer after 2007 compared to before (**Fig. 5A**), irrespective of whether or not the coastal samples were included. The majority of the *Bacteroidetes* sequences belonged to *Flavobacteria* (87%), with *Polaribacter* being the most commonly encountered genus, accounting for one fourth of the *Flavobacteria* OTUs. *Verrucomicrobia* and *Planctomycetes* were often recovered, but no trends were detected. After 2007, unclassified *Proteobacteria*, defined as taxonomically unknown to the RDP classifier and potential novel sequences or non-curated environmental groups, appeared to be more frequent, but this was not statistically significant. Separate BLASTn analysis indicated that some of these were possibly mitochondrial sequences with closest matches to Prasinophytes, especially *Micromonas*, however the similarity was only ∼88% and we could not rule out the possibility of them representing novel bacteria. We found no significant effect of season, PAR, Chl *a* size fraction or geography on the bacterial taxa recovered (**Table 1**).

There were some significant changes for several eukaryotic groups over time (**Fig. 4B**, **5C, S** **6**). After 2007, the proportion of sequences with closest matches to stramenopiles fell significantly, independent of geography or other factors. These sequences had matches mostly to diatoms and uncultured marine heterotrophic flagellates (MASTs [**27**]; **Fig. S5A**). The Chl *a* concentration in the September 2005 sample was 2.14 µg L^−1^, whereas all other samples were below 1 µg L^−1^ (**Fig. 1** **)**. Visually, the sample was dominated by the small diatom *Chaetoceros socialis* (onboard microscopy) and many of the 18S rRNA gene OTUs had best matches to *Chaetoceros socialis* and related species (**Fig. S6**). However, the overall decrease in the proportion of stramenopiles over time was driven primarily by the MASTs. Other groups changed as well, with *Cryothecomonas* (Rhizaria, Cercozoa) significantly less represented after 2007 when Franklin Bay samples were included. Alveolates, mostly small dinoflagellates and ciliates (**Fig. S5B**), were common in all years (**Fig. 4B**), however ciliate sequences, represented primarily by four genera (*Strombidium*, *Novistrombidium*, *Codonellopsis* and *Pseudotontonia*; **Fig. S6**), significantly increased after 2007 (**Fig. 5C**) even when excluding Franklin Bay samples. Haptophytes, with best matches to *Phaeocystis* and *Chrysochromulina*, significantly increased after 2007 when Franklin Bay samples were included. The dominant OTUs (**Fig. S6**) mirrored the whole community, showing a significant overall reduction (*p* = 0.05) in the numbers of stramenopiles and overall increase (*p* = 0.02) of ciliates after 2007. There was no significant trend in the OTU occurrences of *Micromonas* strain CCMP2099, a ubiquitous Arctic ecotype, over time. Similar to the bacterial analysis, among Eukarya we did not detect any significant influences by season, PAR or Chl *a* size distribution (**Table 1**).

Except for 2004, the majority of Archaea OTUs belonged to *Euryarchaeota* Marine Group-II (Eury-MG-II; **Fig. 4C** and **5B**) and they showed a significant linear increase over the 8 years (**Table 1**). Over the same period, the *Thaumarchaeota* MG-I (Thaum-MG-I; formally classified within the *Crenarchaeota*), showed the inverse trend. This striking pattern was repeated amongst the dominant OTUs (**Fig. S7**), where the two Thaum-MG-I OTU groups represented up to ∼60% of all Archaea sequences before 2007 (*p* = 0.04 and 0.05 for decrease). Afterwards, the situation was inversed, with Eury-MG-II OTUs representing 70% of all archaeal sequences in 2010 (*p* = 0.05 for increase). With the exception of a geographic influence, no other significant effects due to the other factors were noted (**Table 1**).

## Discussion

The main bloom in this region occurs in April or May, when surface Chl *a* levels can reach 5 to 12 µg L^−1^ [**28**]. Following the spring bloom, surface waters are depleted in nitrogen and support very low levels of primary productivity [**11]**, [16], [29****]. Although Chl *a* levels are generally below 1 µg L^−1^ in the SCM, this peak in water column biomass is a striking features of much of the oligotrophic Canadian Arctic [**16]**, [30****]. Post-bloom conditions persist though the autumn, but occasional local upwelling and advective nutrient input can promote sporadic increased concentrations of Chl *a* of ca. 2 µg L^−1^ [**31**]. The September 2005 sample was an example of such an event with the lightly silicified diatom *Chaetoceros socialis* appearing in the SCM layer. Similar peaks in bacterial taxa, especially unclassified *Alphaproteobacteria* and *Bacteroidetes*, were also noted suggesting transient conditions can favor select taxa of Bacteria as well as phytoplankton. Similar peaks in specific archaeal taxa may also suggest transient conditions. The goal of our study was to find trends by pooling the data from before and after 2007. Overall significant differences in the probability of different taxa occurring were able to be distinguished from such occasional events.

All three microbial domains showed significant shifts in some species or genera coinciding with the timing of changes in the global environmental conditions. Taxon ordination and OTU similarity trees indicated that diminishing summer ice cover over time was the best indicator of such taxonomic changes. Bacterial diversity fell over time, with fewer OTUs in recent years and the fewer OTUs accounting for higher percentages of the total populations. The numbers of OTUs from the other two domains, in contrast, did not change and storage of the samples over time would not explain the decrease in diversity of Bacteria. In addition, if taxa were lost during storage of extracted DNA, fewer species would be retrieved from the older samples unless substantial degradation artificially increased the number of OTUs. A recent paper by Kalanetra *et al.* [**32**] used DNA collected from the Arctic Ocean in 1997 and reported no significant losses or degradation of the DNA over the 12 year storage period. An ecological-biological explanation for the increase in bacterial evenness and loss of rare taxa may be that increased stability of the physical layer results in less exchange with adjacent water-masses via mixing and a gradual loss of slower growing or less optimally adapted community members over time [**33**], without replacement by new ribotypes [**34**]. Taxonomically, there was a significant shift away from the *Bacteroidetes* represented by over a dozen genera, but with just over half (53%) belonging to two taxa: the psychrophilic *Polaribacter* [**35**] and unidentified *Flavobacteriaceae*. *Bacteroidetes* have a preference for, and selective advantage when growing upon, complex organic matter (OM) [**36**]; additionally *Bacteroidetes* and phytoplankton that produce complex OM exudates [**37**] are often found together [**38]**–[40****]. *Bacteroidetes* are also significant and persistent members of sea ice biota, for example ice-associated *Polaribacter* are able to breakdown and grow on OM in brine channels [**41]**, [42****]. Lo Giudice *et al.* [**43**] suggested that annual melt of sea ice is a source of *Bacteroidetes* found in the water column in spring and summer. Alternatively, Arctic water-column *Bacteroidetes* may be pelagic and populations increase when OM is released by melting sea ice [**40**]. Regardless of the mechanism, the decrease in *Bacteroidetes* OTUs over time suggests that they were an indicator of changes in the OM characteristics in the SCM.

Among the three domains, Archaeal OTUs showed the pattern reflecting the most substantial change concurrent with reduced summer sea ice extent, with significant compositional changes within communities. Taxonomically, there was a decline in Thaum-MG-I over time, albeit with a strong geographic component. This may seem counter-intuitive as Thaum-MG-I have been associated with higher particle loads from river input and with the melting and flux of material from seasonal ice [**44**], which are becoming more prevalent in a warming Arctic. However, since 2007, ice free conditions have arrived earlier in the Amundsen Gulf and the seasonally longer exposure to higher irradiances would create less favorable conditions for the Thaum-MG-I since they are likely inhibited by light [**45**]. Regardless the cause, their loss could have an effect on nitrogen availability to phytoplankton [**45]**, [46****]. Thaum-MG-I oxidize ammonia as an energy source, and as fewer and fewer were available to carry out the first steps that convert ammonia to nitrate over time, their loss may have contributed to the decreasing nitrate concentrations in the SCM. Diatoms are thought to be better competitors for nitrate compared to picophytoplankton [**47]**–[49****] and the loss Thaum-MG-I could have unexpected effects on the capacity of diatoms to persist in the SCM. However, because of our focus on the smaller size fraction, larger robust diatoms were not well sampled and additional work is required to test this notion.

Total eukaryote diversity (indices and OTUs) was much higher than reported from standard cloning and sequencing studies in this same region [**7]**, [50****]. Although we only sequenced the size fraction between 0.2 and 3 microns, it is routine to recover sequences from more flexible or fragile cells that break up during filtration [**50]**, [51****] and the diversity reported here would include these as well as rare OTUs not detected using standard cloning and sequencing approaches. All of the major groups we recovered have been previously reported, and the diversity found here suggests high species-level diversity, especially among small dinoflagellates which group together and are referred to as the Gymnodiniales-Peridiniales-Prorocentrales (GPP) complex [**52**]. These groups represent much of the diversity among Eukarya in most Arctic waters studied to date [**7]**, [50], [53****]. Several other dinoflagellates ribotypes were within clades of heterotrophic and mixotrophic species, notably the *Gyrodinium helveticum* and *G. rubrum* group [**14]**, [54****]. These *Gyrodinium* spp. graze on other protists including phytoplankton [**55]**, [56****] and fluctuations in their relative abundance may be an indication of shifts in the microbial food web dynamics. However, the major changes within the Eukarya sequences came from other heterotrophs; ciliates significantly increased and bacteriovorus MASTs [**27**] decreased, perhaps indicating overall changes in available prey among the Arctic microbiota. After 2007, MASTs may have access to fewer *r*-strategist bacteria, such as the *Bacteroidetes*, which have larger cell-sizes and are preferred targets for bacteriovory. Finally, particular picoeukaryote photosynthetic groups, such as the Haptophytes and the Arctic ecotype of *Micromonas* CCMP2099 tended to increase and suggesting their physiological capacity to thrive over a range of conditions still present in the Arctic [**57**].

The continuing loss of sea ice in the Arctic has been mostly considered in the context of climate forcing and physical feedback. Some modeling results have suggested that the longer open-water season in the Arctic will result in increased productivity [**58]**, [59****]. These models all assume that more open water will lead to increased mixing and entrainment of nutrients into the upper water column. Alternatively, the freshening of the Arctic could lead to stronger salinity stratification and fewer nutrients being entrained into the euphotic zone. The Beaufort Sea-Amundsen Gulf region has been a significant contributor to the Arctic-wide melt of multiyear sea ice [**2**] and the major effect of the ice melt has been a its contribution to the upper Arctic Ocean freshwater budget. The gradual decrease in salinity of the Pacific-origin water is consistent with the overall freshening of the Beaufort and increasing stratification since deep waters below the halocline are not affected. The lack of mixing is also consistent with the drawdown of nitrate over time in the region of the SCM layer of Amundsen Gulf. The SCM occurs at the bottom of the euphotic zone and increasing stratification and nutrient depletion of the upper waters may act to force the SCM deeper resulting in lower ambient PAR for an already light limited community [**30**]. The long-term consequence of further increasing stratification will be reduction of the vertical flux of nutrients to the euphotic zone and deceasing productivity of the entire Beaufort region [**29**]. The Amundsen Gulf itself is a broad (∼200 km), deep (∼500 m) tributary basin of the Canada Basin. Owing to free exchange of water masses above the sill depth (∼350 m), the biophysical properties of its surface waters are broadly representative of the neighboring Canada Basin, where the trends toward increased stratification and a reduction of nitrate since 2002 is evident [**5**]. Biologically, there has been a size shift in the Canada Basin photosynthetic community where small cells have replaced larger species [**5**]. A physical size-shift in Arctic plankton has theoretical downstream effects on nutrient and carbon cycling. Changes in bacterial, archaeal and protist plankton composition will have internal feedbacks and understanding these is a prerequisite to predicting the ecological consequences of continuing climate-forced oceanographic changes. Here we report the first indications of significant changes the occurrence of multiple microbial taxa in the SCM, a biologically significant feature of much of the Western Arctic.

In conclusion, our results point towards a change in the physical structure and nitrate concentrations in the SCM of the Western Arctic and changes in microbial communities over the same time period. Changes in the photosynthetic species composition and microbial food web structure has a direct and substantial effect on zooplankton that can cascade throughout the entire Arctic food chain [**60]**, [61****]. Equally, changes in bacterial and archaeal communities that are responsible for nutrient and carbon biogeochemical cycling [**62**] may have downstream effects on net productivity and the tendency of the Arctic Ocean to sequester or release atmospheric carbon dioxide [**63]**, [64****]. With our approach, we were able to identify the communities in the SCM before and after a major physical change in the Arctic Ocean. As change continues, knowing something not only of the taxonomy, but also of the functional capacity and ecology, of the three microbial domains of life will become critical for predicting the consequences of a warmer, more stratified, and perhaps soon-to-be seasonally ice-free Arctic.

## Supporting Information

Figure S1 **Rarefaction analysis for Bacteria (97% OTU level), Eukaryotes (98% OTU level) and Archaea (97% OTU level).** The inset box shows the Chao1 total richness estimate for the three domains.
(PDF)

Figure S2 **Whole community diversity comparisons.** Correspondence analysis calculated from the percent taxonomic distributions of the total OTUs. Dots are colored according to Ice Extent: <2007 in blue and >2007 in red. (**A**) Bacteria 97% phylum-level OTUs. (**B**) Eukarya 98% major-group-level OTUs. (**C**) Archaea 97% genus-level OTUs.
(PDF)

Figure S3 **Whole community diversity comparisons.** Bray-Curtis similarity trees were calculated from total OTU compositions (independent of taxonomy). Trees are colored according to Ice Extent: <2007 in blue and >2007 in red. (**A**) Bacteria 97% level OTUs. (**B**) Eukarya 98% level OTUs. (**C**) Archaea 97% level OTUs.
(PDF)

Figure S4 **Taxonomic distribution of the top 50 dominant Bacteria OTUs (97% level).** Colors are as in Figure 4: *α-Proteobacteria* (AlphaP) are in dark purple, *γ-Proteobacteria* (GammaP) in light purple, *δ-Proteobacteria* (DeltaP) in rose; *Bacteroidetes* in orange, *Verrucomicrobia* (Verruco) in red, *Planctomycetes* (Plancto) in blue and unclassified (unclass.) sequences are in black. Rhodobact., *Rhodobacteraceae*; Rhodospiril., *Rhodospirillaceae*; AlteroO, *Alteromonadales*; OceanoO, *Oceanospirillales*; CystoF, *Cystobacteraceae*; Cryo., *Cryomorphaceae*; FlavoF, *Flavobacteriaceae*. Gray squares indicate complete absence (0%).
(PDF)

Figure S5 **Taxonomic distributions of select Eukarya group OTUs (98% level).** (**A**) Distribution of stramenopiles. MAST, uncultured marine stramenopile groups. (**B**) Distribution of Alveolates. MALV, uncultured marine Alveolate groups.
(PDF)

Figure S6 **Taxonomic distribution of the top 50 dominant eukaryotic OTUs (98% level).** Colors are as in Figure 4: the different sub-groups of Alveolates are in shades of blue, the Biliphytes in pink, the Choanoflagellates (Choano) in orange, the Haptophytes in purple, the Prasinophytes (Prasino) in green, the Rhizaria in yellow, the stramenopile sub-groups in shades of red and the unclassified (unclass.) levels in black. Intramacro., Intramacronucleata; DinoGPP, *Gymnodinium-Peridinium-Prorocentrum* complex; Environ, environmental; MALV, uncultured marine Alveolate group; MAST, uncultured marine stramenopile group. Gray squares indicate complete absence (0%).
(PDF)

Figure S7 **Taxonomic distribution of the top 50 dominant Archaea OTUs (97% level).** Colors are as in Figure 4: *Thaumarchaeota* (ThaumA) Marine Group (MG) I are in shades of blue and *Euryarchaeota* (EuryA) MG-II in red.
(PDF)

Table S1 16S/18S rDNA primer re-design (original primers are on gray lines, new on white).
(PDF)

Table S2 16/18S pyrosequencing primers.
(PDF)

Table S3 MIMARKS-compliant table of the pyrosequencing samples.
(XLS)

Table S4 Pyrotag raw data, filtering and OTU statistics.
(PDF)

Table S5 Assignments of the samples to the various categories used to test for significant trends (see Fig. 1 and Table 1).
(PDF)
