# Supplementary material for: Arctic Ocean Microbial Community Structure before and after the 2007 Record Sea Ice Minimum
Source: PLoS One. 2011 Nov 9;6(11):e27492. doi: 10.1371/journal.pone.0027492 (PMC3212577; doi:10.1371/journal.pone.0027492)
Supplement: Figure S1 — Rarefaction analysis for Bacteria (97% OTU level), Eukaryotes (98% OTU level) and Archaea (97% OTU level). The inset box shows the Chao1 total richness estimate for the three domains. (PDF) [file pone.0027492.s001.pdf]

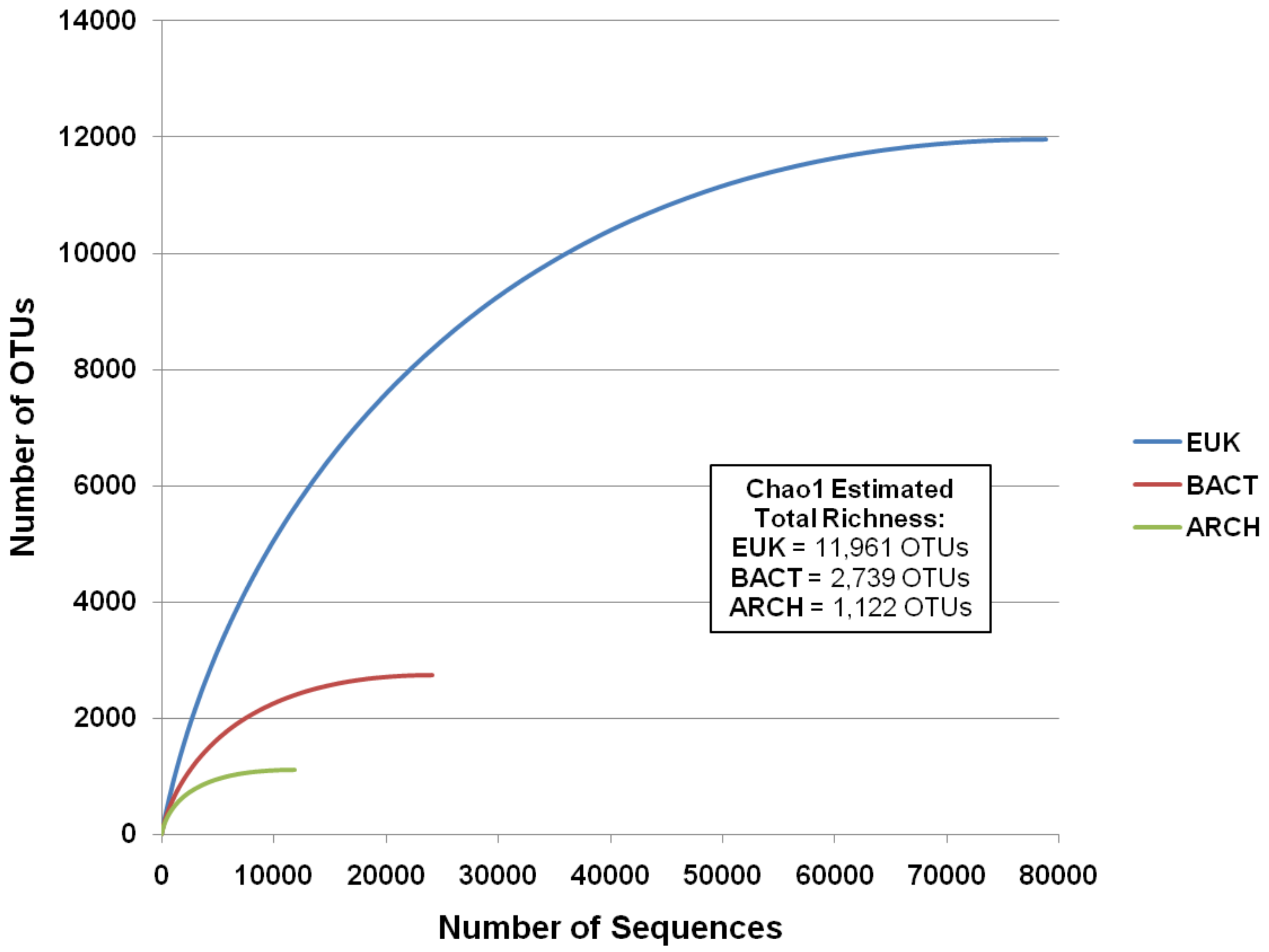

Figure S1. Rarefaction analysis for Bacteria (97% OTU level), Eukaryotes (98% OTU level) and Archaea (97% OTU level). The inset box shows the Chao1 total richness estimate for the three Domains.
