# Supplementary material for: Arctic Ocean Microbial Community Structure before and after the 2007 Record Sea Ice Minimum
Source: PLoS One. 2011 Nov 9;6(11):e27492. doi: 10.1371/journal.pone.0027492 (PMC3212577; doi:10.1371/journal.pone.0027492)
Supplement: Figure S2 — Whole community diversity comparisons. Correspondence analysis calculated from the percent taxonomic distributions of the total OTUs. Dots are colored according to Ice Extent: <2007 in blue and >2007 in red. (A) Bacteria 97% phylum-level OTUs. (B) Eukarya 98% major-group-level OTUs. (C) Archaea 97% genus-level OTUs. (PDF) [file pone.0027492.s002.pdf]

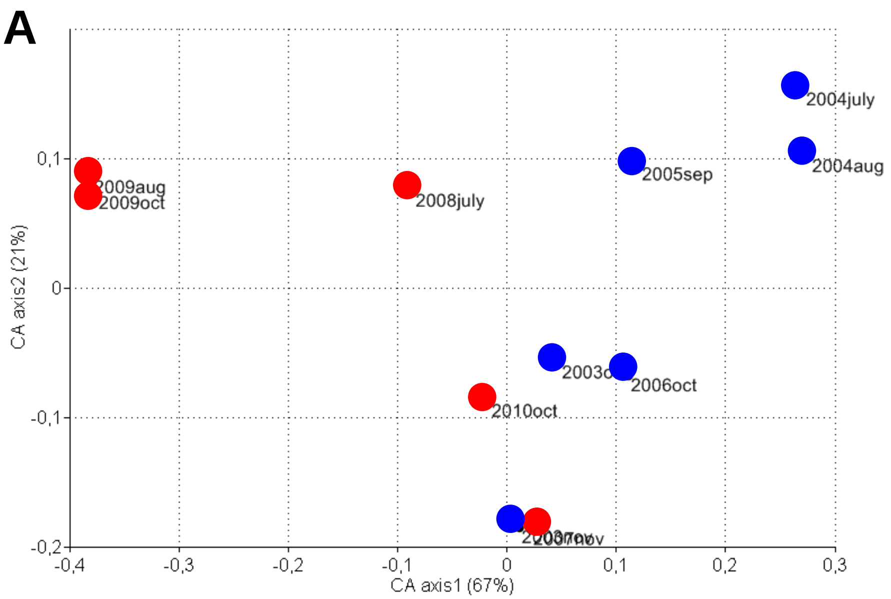

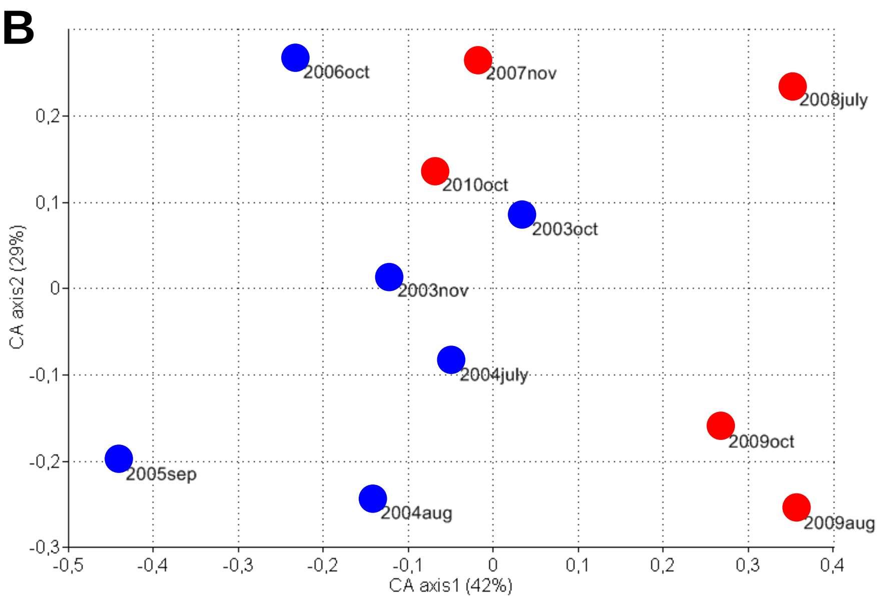

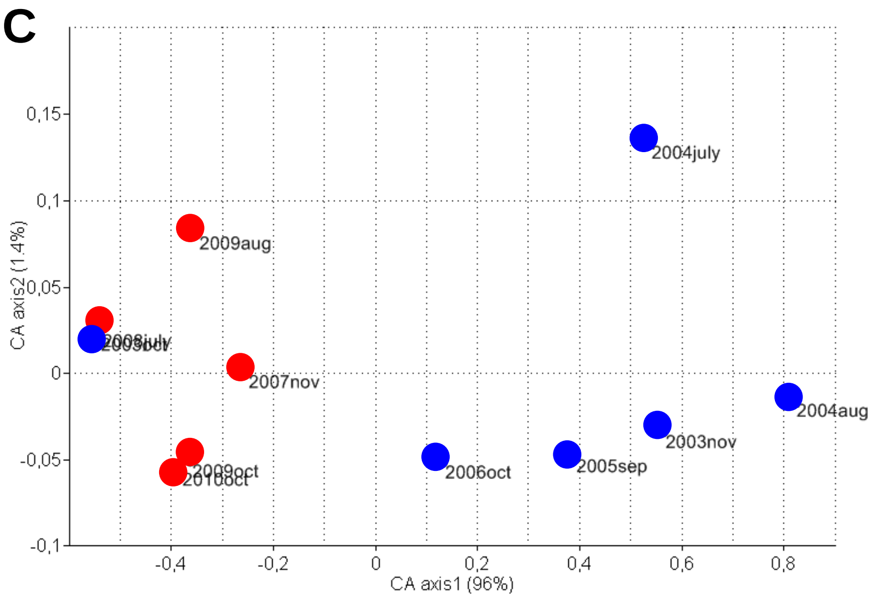

Figure S2. Whole community diversity comparisons. Correspondence analysis calculated from the percent taxonomic distributions of the total OTUs. Dots are colored according to Ice Extent: <2007 in blue and >2007 in red. (**A**) Bacteria 97% phylum-level OTUs. (**B**) Eukarya 98% major-group-level OTUs. (**C**) Archaea 97% genus-level OTUs.
