# Supplementary material for: Arctic Ocean Microbial Community Structure before and after the 2007 Record Sea Ice Minimum
Source: PLoS One. 2011 Nov 9;6(11):e27492. doi: 10.1371/journal.pone.0027492 (PMC3212577; doi:10.1371/journal.pone.0027492)
Supplement: Figure S4 — Taxonomic distribution of the top 50 dominant Bacteria OTUs (97% level). Colors are as in Figure 4: α-Proteobacteria (AlphaP) are in dark purple, γ-Proteobacteria (GammaP) in light purple, δ-Proteobacteria (DeltaP) in rose; Bacteroidetes in orange, Verrucomicrobia (Verruco) in red, Planctomycetes (Plancto) in blue and unclassified (unclass.) sequences are in black. Rhodobact., Rhodobacteraceae; Rhodospiril., Rhodospirillaceae; AlteroO, Alteromonadales; OceanoO, Oceanospirillales; CystoF, Cystobacteraceae; Cryo., Cryomorphaceae; FlavoF, Flavobacteriaceae. Gray squares indicate complete absence (0%). (PDF) [file pone.0027492.s004.pdf]

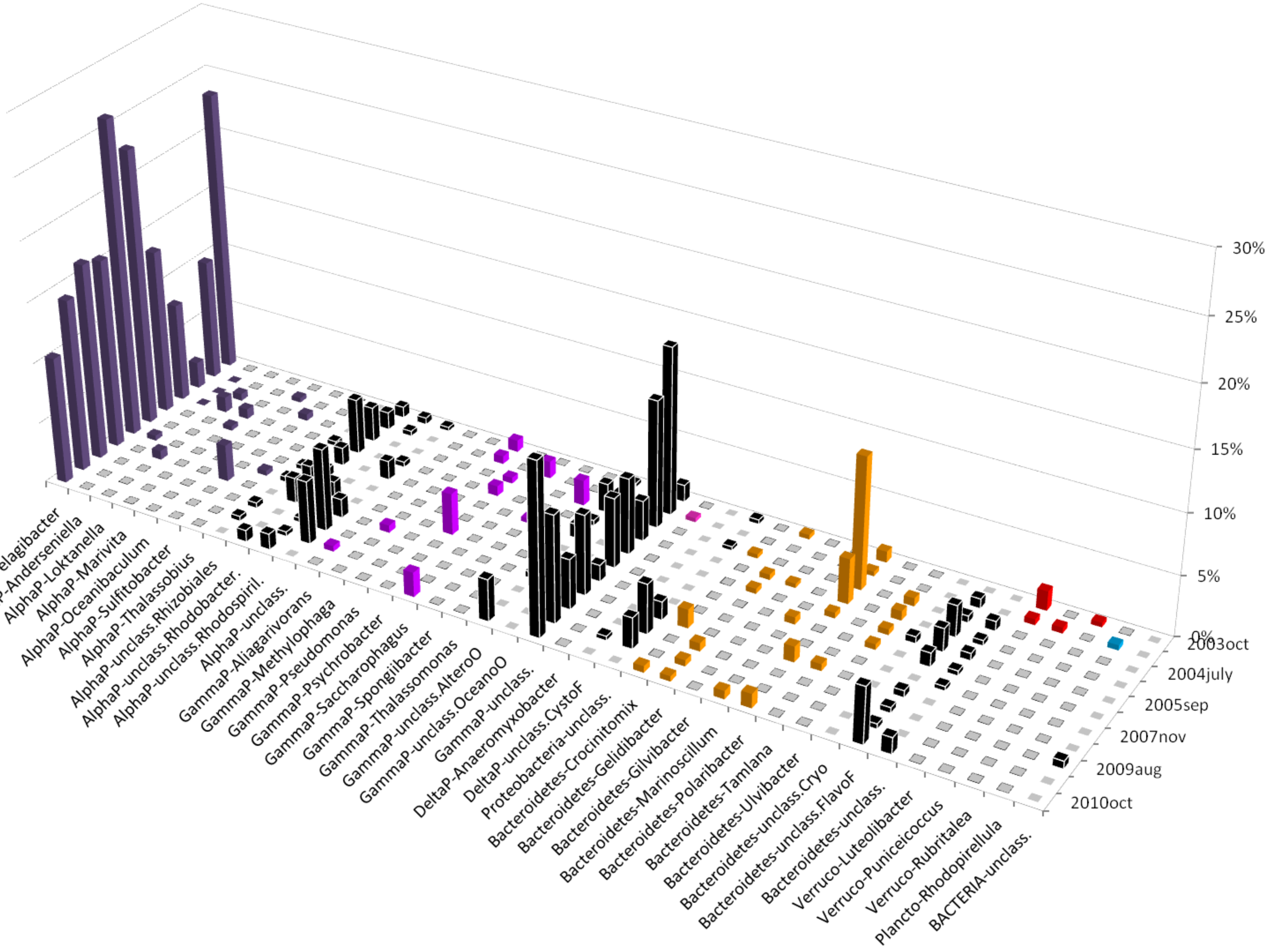

Figure S4. Taxonomic distribution of the top 50 dominant Bacteria OTUs (97% level). Colors are as in Figure 4: *α-Proteobacteria* (AlphaP) are in dark purple, *γ-Proteobacteria* (GammaP) in light purple, *δ-Proteobacteria* (DeltaP) in rose; *Bacteroidetes* in orange, *Verrucomicrobia* (Verruco) in red, *Planctomycetes* (Plancto) in blue and unclassified (unclass.) sequences are in black. Rhodobact., *Rhodobacteraceae*; Rhodospiril., *Rhodospirillaceae*; AlteroO, *Alteromonadales*; OceanoO, *Oceanospirillales*; CystoF, *Cystobacteraceae*; Cryo., *Cryomorphaceae*; FlavoF, *Flavobacteriaceae*. Gray squares indicate complete absence (0%). Note that plastid sequences are excluded.
