# Supplementary material for: Arctic Ocean Microbial Community Structure before and after the 2007 Record Sea Ice Minimum
Source: PLoS One. 2011 Nov 9;6(11):e27492. doi: 10.1371/journal.pone.0027492 (PMC3212577; doi:10.1371/journal.pone.0027492)
Supplement: Figure S5 — Taxonomic distributions of select Eukarya group OTUs (98% level). (A) Distribution of stramenopiles. MAST, uncultured marine stramenopile groups. (B) Distribution of Alveolates. MALV, uncultured marine Alveolate groups. (PDF) [file pone.0027492.s005.pdf]

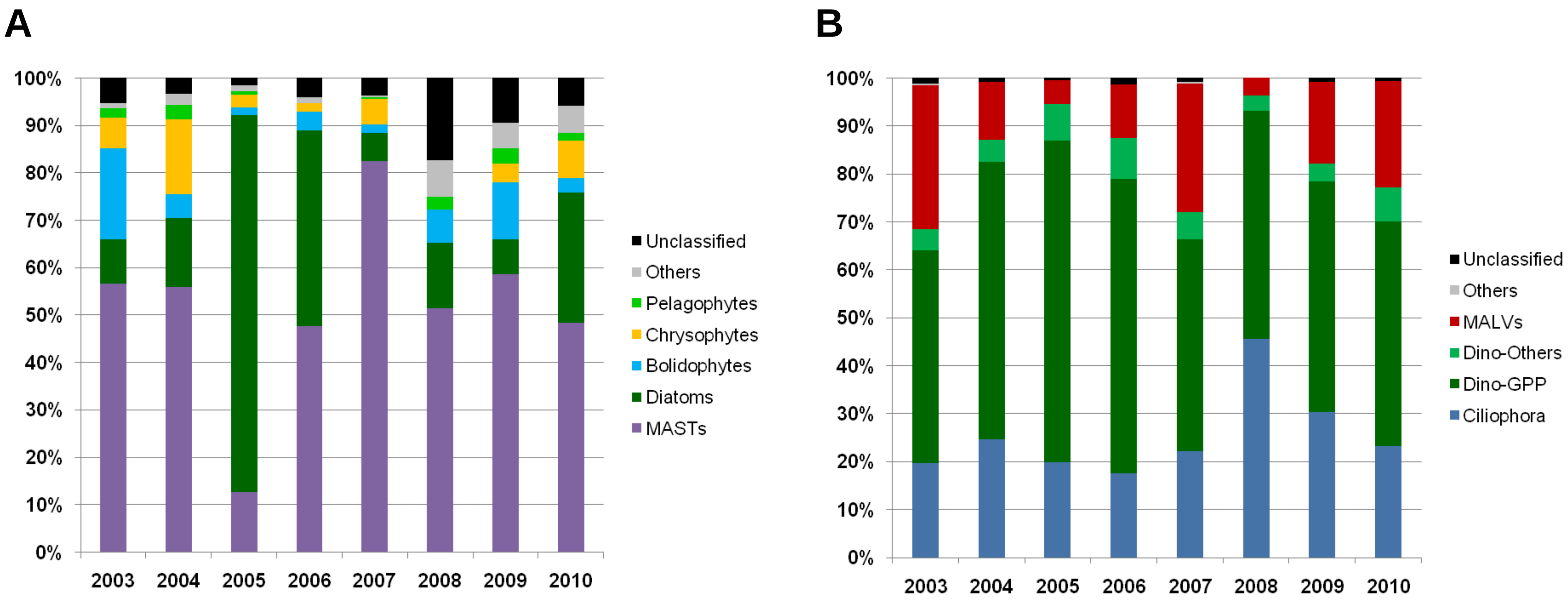

Figure S5. Taxonomic distributions of select Eukarya group OTUs (98% level). (**A**) Distribution of stramenopiles. MAST, uncultured marine stramenopile groups. (**B**) Distribution of Alveolates. MALV, uncultured marine Alveolate groups.
