# Supplementary material for: Arctic Ocean Microbial Community Structure before and after the 2007 Record Sea Ice Minimum
Source: PLoS One. 2011 Nov 9;6(11):e27492. doi: 10.1371/journal.pone.0027492 (PMC3212577; doi:10.1371/journal.pone.0027492)
Supplement: Figure S6 — Taxonomic distribution of the top 50 dominant eukaryotic OTUs (98% level). Colors are as in Figure 4: the different sub-groups of Alveolates are in shades of blue, the Biliphytes in pink, the Choanoflagellates (Choano) in orange, the Haptophytes in purple, the Prasinophytes (Prasino) in green, the Rhizaria in yellow, the stramenopile sub-groups in shades of red and the unclassified (unclass.) levels in black. Intramacro., Intramacronucleata; DinoGPP, Gymnodinium-Peridinium-Prorocentrum complex; Environ, environmental; MALV, uncultured marine Alveolate group; MAST, uncultured marine stramenopile group. Gray squares indicate complete absence (0%). (PDF) [file pone.0027492.s006.pdf]

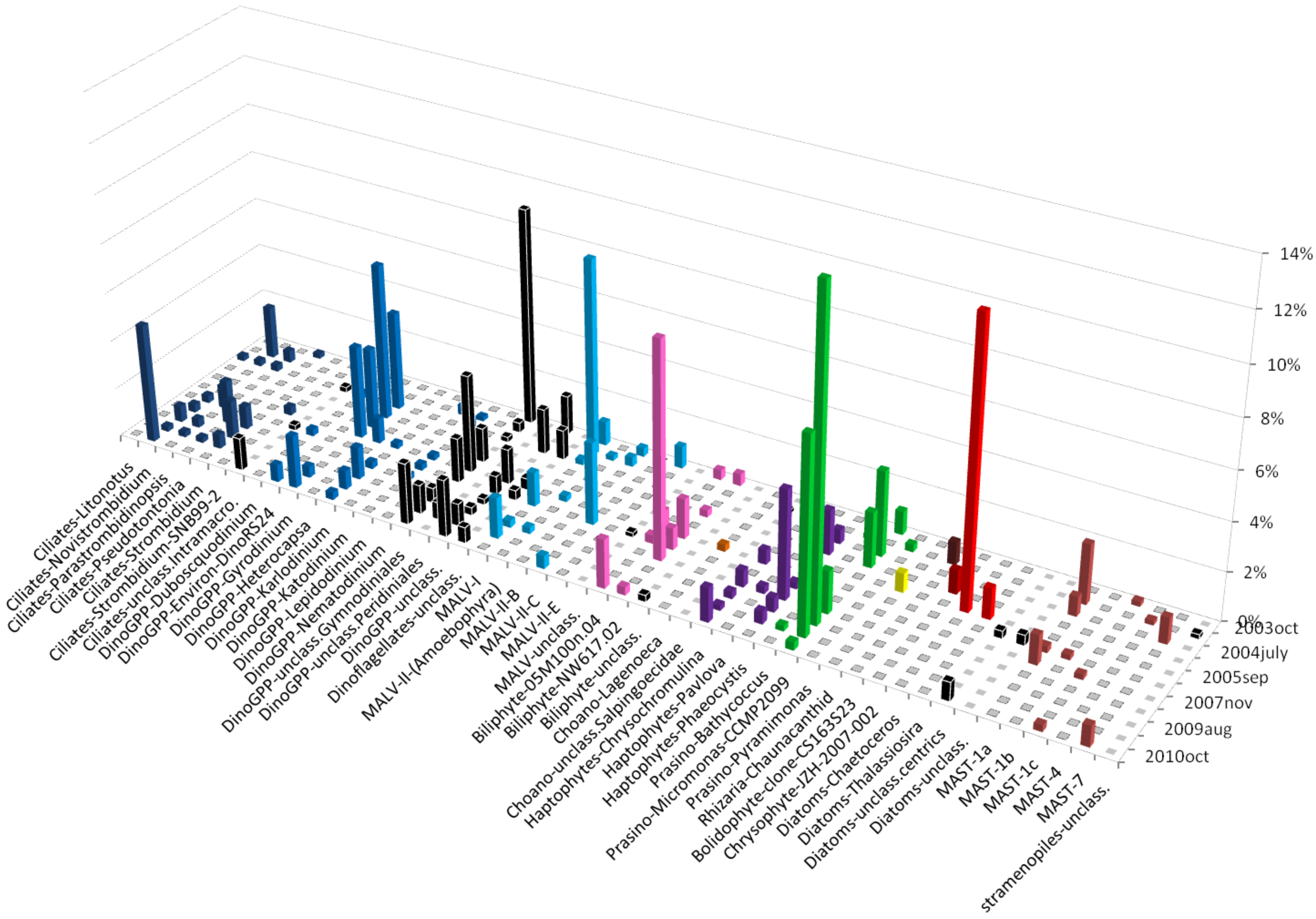

Figure S6. Taxonomic distribution of the top 50 dominant eukaryotic OTUs (98% level). Colors are as in Figure 4: the different sub-groups of Alveolates are in shades of blue, the Biliphytes in pink, the Choanoflagellates (Choano) in orange, the Haptophytes in purple, the Prasinophytes (Prasino) in green, the Rhizaria in yellow, the stramenopile sub-groups in shades of red and the unclassified (unclass.) levels in black. Intramacro., Intramacronucleata; DinoGPP, *Gymnodinium-Peridinium-Prorocentrum* complex; Environ, environmental; MALV, uncultured marine Alveolate group; MAST, uncultured marine stramenopile group. Gray squares indicate complete absence (0%).
