# Supplementary material for: Arctic Ocean Microbial Community Structure before and after the 2007 Record Sea Ice Minimum
Source: PLoS One. 2011 Nov 9;6(11):e27492. doi: 10.1371/journal.pone.0027492 (PMC3212577; doi:10.1371/journal.pone.0027492)
Supplement: Figure S7 — Taxonomic distribution of the top 50 dominant Archaea OTUs (97% level). Colors are as in Figure 4: Thaumarchaeota (ThaumA) Marine Group (MG) I are in shades of blue and Euryarchaeota (EuryA) MG-II in red. (PDF) [file pone.0027492.s007.pdf]

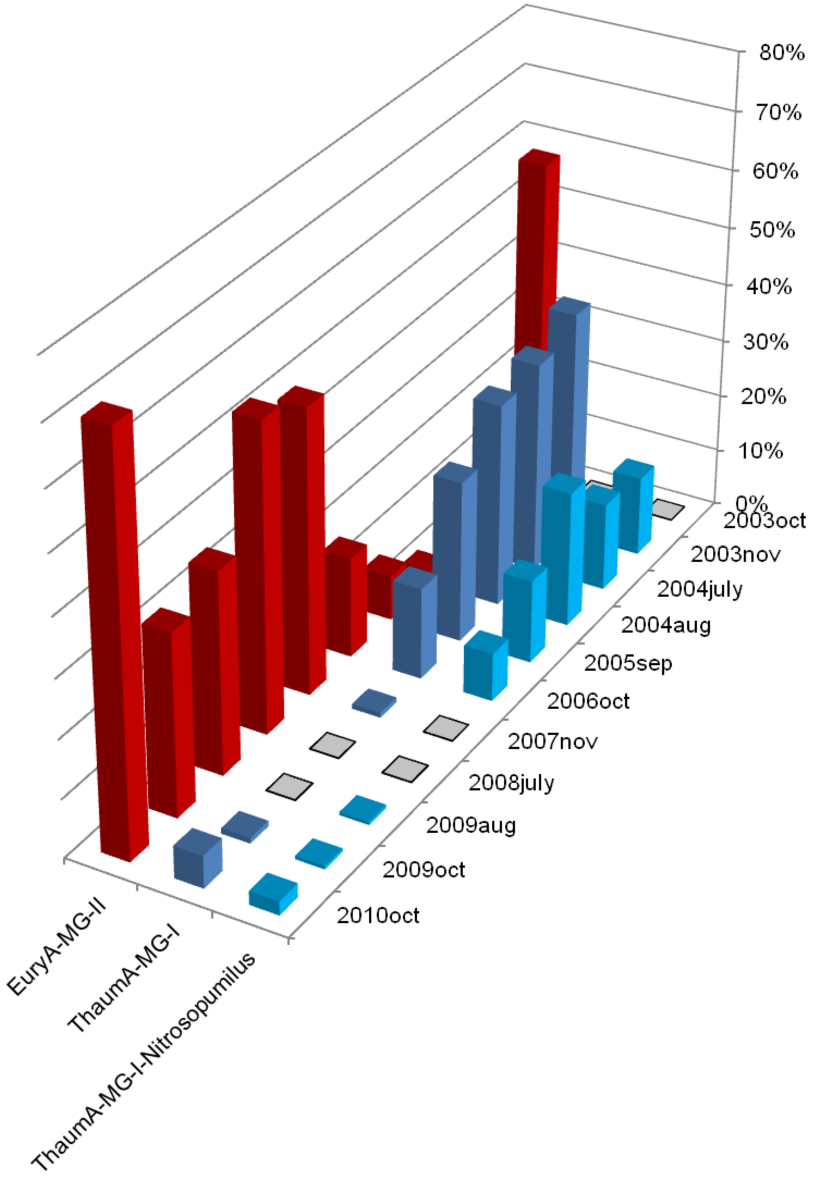

Figure S7. Taxonomic distribution of the top 50 dominant Archaea OTUs (97% level). Colors are as in Figure 4: *Thaumarchaeota* (ThaumA) Marine Group (MG) I are in shades of blue and *Euryarchaeota* (EuryA) MG-II in red. Gray squares indicate complete absence (0%).
