# Supplementary material for: Arctic Ocean Microbial Community Structure before and after the 2007 Record Sea Ice Minimum
Source: PLoS One. 2011 Nov 9;6(11):e27492. doi: 10.1371/journal.pone.0027492 (PMC3212577; doi:10.1371/journal.pone.0027492)
Supplement: Table S1 — 16S/18S rDNA primer re-design (original primers are on gray lines, new on white). (PDF) [file pone.0027492.s008.pdf]

Table S1. 16S/18S rDNA primer re-design (original primers are on gray lines, new on white).

Name	16S/18S Sequence 5'-3'	T_m	range	RDP Bacteria specificity (with X mismatches) *n*	0	1	2	RDP Archaea specificity (with X mismatches) *n*	0	1	2
B967Fmix[a]	MNA MSC GMN RAA CCT YAN C	55	44-65	652,300	90%	98%	99%	18,845	-	-	25%
**B969F**	**A CGC GHN RAA CCT TAC C**	**53**	**49-58**	**652,300**	**92%**	**98%**	**99%**	**18,845**	**-**	**-**	**18%**
BA1406R[b]	AC GGG CGG TGW GTR CAA	59	57-61	331,093	96%	99%	~100%	6,756	94%	98%	98%
**BA1406R**	**AC GGG CRG TGW GTR CAA**	**57**	**53-61**	**331,093**	**97%**	**99%**	**~100%**	**6,756**	**95%**	**98%**	**98%**
A958F[a]	AAT TGG ANT CAA CGC CGG	55	54-56	653,739	-	-	-	19,230	69%	92%	95%
**A956F**	**TY AAT YGG ANT CAA CRC C**	**50**	**45-55**	**653,739**	**-**	**-**	**-**	**19,230**	**87%**	**94%**	**97%**
**A1401R**	**CRG TGW GTR CAA GGR GCA**	**56**	**51-62**	**327,184**	**-**	**-**	**-**	**8,401**	**90%**	**96%**	**97%**
Euk528F[b]	CC GCG GTA ATT CCA GCT CcA a	60	-	1,227,352	-	-	-	53,616	-	-	-
**E572F**	**CY GCG GTA ATT CCA GCT C**	**55**	**53-56**	**1,227,352**	**-**	**-**	**-**	**53,616**	**-**	**8%**	**25%**
Brate R1[c]	CgA AGA YGA TYA GAT ACC gT	51	49-54	**1,227,352**	**-**	**-**	**-**	**53,616**	**-**	**-**	**-**
**E1009R**	**CRA AGA YGA TYA GAT ACC RT**	**49**	**44-54**	**1,227,352**	**-**	**-**	**-**	**53,616**	**-**	**-**	**-**

Legend: A – nucleotide <95% consensus; a – nucleotide <90% consensus; A – nucleotide mismatch vs. *E. coli*; X% – 95% or more RDP specificity; X% – 80-94% RDP specificity; X% – <80% RDP specificity

Designed using ARB (http://www.arb-home.de/) with the SILVA 100-SSU-Ref database (http://www.arb-silva.de/) containing: 20,854 Eukarya (ignoring metazoans/fungi), 17,127 Archaea, and 345,332 Bacteria. T_m calculated using the IDT OligoAnalyzer (http://www.idtdna.com/analyzer/Applications/OligoAnalyzer/). New Eukarya primers utilize the *S. cerevisiae* 18S numbering.

[a] The consensus of the F primer mix for 454 pyrosequencing used by VAMPS (http://vamps.mbl.edu/resources/primers.php).

[b] Standard 16/18S primers [1-3].

[c] 454 pyrosequencing R primer from Bråte *et al.* [4].

1. Baker G, Smith JJ, Cowan DA (2003) Review and re-analysis of domain-specific 16S primers. J Microbiol Meth 55: 541-555.
2. Romari K, Vaulot D (2004) Composition and temporal variability of picoeukaryote communities at a coastal site of the English Channel from 18S rDNA sequences. Limnol Oceanogr 49: 784-798.
3. Zhu F, Massana R, Not F, Marie D, Vaulot D (2005) Mapping of picoeukaryotes in marine ecosystems with quantitative PCR of the 18S rRNA gene. FEMS Microbiol Ecol 52: 79-92.
4. Bråte J *et al.* (2010) Freshwater Perkinsea and marine-freshwater colonizations revealed by pyrosequencing and phylogeny of environmental rDNA. ISME J 4: 1144-1153.
