# Supplementary material for: Arctic Ocean Microbial Community Structure before and after the 2007 Record Sea Ice Minimum
Source: PLoS One. 2011 Nov 9;6(11):e27492. doi: 10.1371/journal.pone.0027492 (PMC3212577; doi:10.1371/journal.pone.0027492)
Supplement: Table S2 — 16/18S pyrosequencing primers. (PDF) [file pone.0027492.s009.pdf]

Table S2. 16/18S pyrosequencing primers.

Primer Name[a]	Sequence 5'-3'
Bacteria 16S	
A1-B969F	CCATCTCATCCCTGCGTGTCTCCGACTCAG•ACGAGTGCGT•ACGCGHNRAACCTTACC
A2-B969F	CCATCTCATCCCTGCGTGTCTCCGACTCAG•ACGCTCGACA•ACGCGHNRAACCTTACC
A3-B969F	CCATCTCATCCCTGCGTGTCTCCGACTCAG•AGACGCACTC•ACGCGHNRAACCTTACC
A4-B969F	CCATCTCATCCCTGCGTGTCTCCGACTCAG•AGCACTGTAG•ACGCGHNRAACCTTACC
A5-B969F	CCATCTCATCCCTGCGTGTCTCCGACTCAG•ATCAGACACG•ACGCGHNRAACCTTACC
A6-B969F	CCATCTCATCCCTGCGTGTCTCCGACTCAG•ATATCGCGAG•ACGCGHNRAACCTTACC
A7-B969F	CCATCTCATCCCTGCGTGTCTCCGACTCAG•CGTGTCTCTA•ACGCGHNRAACCTTACC
A8-B969F	CCATCTCATCCCTGCGTGTCTCCGACTCAG•CTCGCGTGTC•ACGCGHNRAACCTTACC
A9-B969F	CCATCTCATCCCTGCGTGTCTCCGACTCAG•TAGTATCAGC•ACGCGHNRAACCTTACC
A10-B969F	CCATCTCATCCCTGCGTGTCTCCGACTCAG•TCTCTATGCG•ACGCGHNRAACCTTACC
A12-B969F	CCATCTCATCCCTGCGTGTCTCCGACTCAG•TACTGAGCTA•ACGCGHNRAACCTTACC
B-BA1406R	CCTATCCCCTGTGTGCCTTGGCAGTCTCAG•ACGGGCRGTGWGTRCAA
Archaea 16S	
A1-A956F	CCATCTCATCCCTGCGTGTCTCCGACTCAG•ACGAGTGCGT•TYAATYGGANTCAACRCC
A2-A956F	CCATCTCATCCCTGCGTGTCTCCGACTCAG•ACGCTCGACA•TYAATYGGANTCAACRCC
A3-A956F	CCATCTCATCCCTGCGTGTCTCCGACTCAG•AGACGCACTC•TYAATYGGANTCAACRCC
A4-A956F	CCATCTCATCCCTGCGTGTCTCCGACTCAG•AGCACTGTAG•TYAATYGGANTCAACRCC
A5-A956F	CCATCTCATCCCTGCGTGTCTCCGACTCAG•ATCAGACACG•TYAATYGGANTCAACRCC
A6-A956F	CCATCTCATCCCTGCGTGTCTCCGACTCAG•ATATCGCGAG•TYAATYGGANTCAACRCC
A7-A956F	CCATCTCATCCCTGCGTGTCTCCGACTCAG•CGTGTCTCTA•TYAATYGGANTCAACRCC
A8-A956F	CCATCTCATCCCTGCGTGTCTCCGACTCAG•CTCGCGTGTC•TYAATYGGANTCAACRCC
A9-A956F	CCATCTCATCCCTGCGTGTCTCCGACTCAG•TAGTATCAGC•TYAATYGGANTCAACRCC
A10-A956F	CCATCTCATCCCTGCGTGTCTCCGACTCAG•TCTCTATGCG•TYAATYGGANTCAACRCC
A12-A956F	CCATCTCATCCCTGCGTGTCTCCGACTCAG•TACTGAGCTA•TYAATYGGANTCAACRCC
B-A1401R	CCTATCCCCTGTGTGCCTTGGCAGTCTCAG•CRGTGWGTRCAAGGRGCA

Table S2 *continued…*

Primer Name[a]	Sequence 5'-3'
Eukarya 18S	
A1-E572F	CCATCTCATCCCTGCGTGTCTCCGACTCAG•ACGAGTGCGT•CYGCGGTAATTCCAGCTC
A2-E572F	CCATCTCATCCCTGCGTGTCTCCGACTCAG•ACGCTCGACA•CYGCGGTAATTCCAGCTC
A3-E572F	CCATCTCATCCCTGCGTGTCTCCGACTCAG•AGACGCACTC•CYGCGGTAATTCCAGCTC
A4-E572F	CCATCTCATCCCTGCGTGTCTCCGACTCAG•AGCACTGTAG•CYGCGGTAATTCCAGCTC
A5-E572F	CCATCTCATCCCTGCGTGTCTCCGACTCAG•ATCAGACACG•CYGCGGTAATTCCAGCTC
A6-E572F	CCATCTCATCCCTGCGTGTCTCCGACTCAG•ATATCGCGAG•CYGCGGTAATTCCAGCTC
A7-E572F	CCATCTCATCCCTGCGTGTCTCCGACTCAG•CGTGTCTCTA•CYGCGGTAATTCCAGCTC
A8-E572F	CCATCTCATCCCTGCGTGTCTCCGACTCAG•CTCGCGTGTC•CYGCGGTAATTCCAGCTC
A9-E572F	CCATCTCATCCCTGCGTGTCTCCGACTCAG•TAGTATCAGC•CYGCGGTAATTCCAGCTC
A10-E572F	CCATCTCATCCCTGCGTGTCTCCGACTCAG•TCTCTATGCG•CYGCGGTAATTCCAGCTC
A12-E572F	CCATCTCATCCCTGCGTGTCTCCGACTCAG•TACTGAGCTA•CYGCGGTAATTCCAGCTC
B-E1009R	CCTATCCCCTGTGTGCCTTGGCAGTCTCAG•AYGGTATCTRATCRTCTTYG

[a] Bar-code #11 was skipped and the 11th sample used Roche MID #12 (i.e.: A12-B969F).

Primers are constituted of 30 bp Roche 454 adaptors (A for forward, B for reverse), 10 bp Roche MID bar-codes (shown between dots for clarity; absent from reverse primers), and the 17-20 bp rDNA-specific sequences detailed in **Table S1**.
