# Supplementary material for: Arctic Ocean Microbial Community Structure before and after the 2007 Record Sea Ice Minimum
Source: PLoS One. 2011 Nov 9;6(11):e27492. doi: 10.1371/journal.pone.0027492 (PMC3212577; doi:10.1371/journal.pone.0027492)
Supplement: Table S4 — Pyrotag raw data, filtering and OTU statistics. (PDF) [file pone.0027492.s011.pdf]

Table S4. Pyrotag raw data, filtering and OTU statistics.

	**Bacteria**	**Eukarya**	**Archaea**
Raw Data[a]			
reads	74,566	173,549	93,040
total sequence	22.0 Mb	63.7 Mb	33.7 Mb
mean length	296 bp	367 bp	362 bp
Quality Filters[b]			
reads with Ns	2,352 (3%)	4,829 (3%)	6,210 (7%)
reads <150 bp	21,791 (29%)	17,632 (10%)	5,762 (6%)
reads longer than expected	9 (<0.1%)	0	0
reads with bad Forward primer	6,127 (8%)	5,674 (3%)	6,293 (7%)
total	30,279 (41%)	28,135 (16%)	18,265 (20%)
Post-Filtering			
final reads	44,287 (69%)	145,404 (84%)	74,775 (80%)
mean length	386 bp	402 bp	382 bp
median length	430 bp	434 bp	426 bp
OTU Analysis[c]			
OTUs (all samples) – non-redundant[d]	2,739	11,961	1,122
OTUs (all samples) – cumulative[e]	9,298	30,405	4,064
mean OTUs (per sample)	845	2,764	369
range OTUs (per sample)	690-937	1,876-3,094	205-503

[a] Raw data for Bacteria was generated at the McGill University/Génome Québec Innovation Centre and for Eukarya/Archaea at the IBIS/Université Laval Plate-forme d'Analyses Génomiques.

[b] Filters were added sequentially, in the presented order; therefore some reads may have fallen into multiple categories.

[c] OTUs were clustered at ≥97% similarity for Bacteria/Archaea and ≥98% for Eukarya. The number of input reads for each bar-code was also re-sampled to have even numbers in all bar-codes (i.e.: equal to the lowest number present in any one bar-code): 2474/sample for Bacteria, 8700/sample for Eukarya and 1118/sample for Archaea.

[d] Non-redundant totals when all reads are clustered together.

[e] Cumulative totals when OTUs are split for each of the 11 bar-codes.
