# Supplementary material for: Arctic Ocean Microbial Community Structure before and after the 2007 Record Sea Ice Minimum
Source: PLoS One. 2011 Nov 9;6(11):e27492. doi: 10.1371/journal.pone.0027492 (PMC3212577; doi:10.1371/journal.pone.0027492)
Supplement: Table S5 — Assignments of the samples to the various categories used to test for significant trends (see Fig. 1 and Table 1). (PDF) [file pone.0027492.s012.pdf]

Table S5. Assignments of the samples to the various categories used to test for significant trends (see Fig. 1 and Table 1).

Sample#	Date	Analysis Factor					
		Ice Extent	Geography	Year	Season	PAR[a]	Chl *a* Distribution[b]
1	Oct.2003	<2007	Open Ocean	2003	Fall	Low	Shared
2	Nov.2003	<2007	Franklin Bay	2003	Fall	Low	Shared
3	July 2004	<2007	Franklin Bay	2004	Summer	Med	Shared
4	Aug.2004	<2007	Franklin Bay	2004	Summer	High	Nano
5	Sep.2005	<2007	Open Ocean	2005	Summer	High	Nano
6	Oct.2006	<2007	Open Ocean	2006	Fall	Med	Nano
7	Nov.2007	>2007	Open Ocean	2007	Fall	Low	Nano
8	July 2008	>2007	Open Ocean	2008	Summer	Med	Pico
9	Aug.2009	>2007	Open Ocean	2009	Summer	High	Pico
10	Oct.2009	>2007	Open Ocean	2009	Fall	Low	Pico
11	Oct.2010	>2007	Open Ocean	2010	Fall	Low	Nano

[a] Low-light was considered as <0.1 µE m^{-2} s^{-1}, medium-light as 0.1-1.0 µE m^{-2} s^{-1} and high-light as >1.0 µE m^{-2} s^{-1} PAR (Photosynthetically-Active Radiation) as measured at the depth sampled (see Fig.1).

[b] The percent distribution of Chlorophyll *a* in the small (<3 µm) vs. large (>3 µm) fractions was taken as a proxy for successional stage. Communities were binned as >60% (Nano-dominated), 40-60% (shared), or <40% (Pico-dominated) in the large fraction as measured at the depth sampled (see Fig.1).
